# Metacognition Beliefs and General Health in Predicting Alexithymia in Students

**DOI:** 10.5539/gjhs.v8n2p117

**Published:** 2015-06-11

**Authors:** Samaneh Babaei, Shahryar Ranjbar Varandi, Zohre Hatami, Maryam Gharechahi

**Affiliations:** 1 Deputy of Research and Technology, Zahedan University of Medical Sciences, Zahedan, IR Iran; 2 Training and Education Organization of Sari, Sari, IR Iran; 3 Department of Clinical Psychology, Sari Branch, Islamic Azad University, Sari, IR Iran; 4 Training and Education Organization of Daregaz, Daregaz, IR Iran; 5 Imam Khomeini Relief Foundation, Neyriz, IR Iran

**Keywords:** alexithymia, cognition, emotions, general health, students

## Abstract

**Objectives:**

The present study was conducted to investigate the role of metacognition beliefs and general health in alexithymia in Iranian students.

**Methods:**

This descriptive and correlational study included 200 participants of high schools students, selected randomly from students of two cities (Sari and Dargaz), Iran. Metacognitive Strategies Questionnaire (MCQ-30); the General Health Questionnaire (GHQ) and Farsi Version of the Toronto Alexithymia Scale (TAS-20) were used for gathering the data. Using the Pearson’s correlation method and regression, the data were analyzed.

**Results:**

The findings indicated significant positive relationships between alexithymia and all subscales of general health. The highest correlation was between alexithymia and anxiety subscale (r=0.36, P<0.01). Also, there was a significant negative relationship between alexithymia and some metacognitive strategies. The highest significant negative relationship was seen between alexithymia and the sub-scale of risk uncontrollability (r=-0.359, P < 0.01). Based on the results of multiple regressions, three predictors explained 21% of the variance (R^2^=0. 21, F=7.238, P<0.01). It was found that anxiety subscale of General Health significantly predicted 13% of the variance of alexithymia (β=0.36, P<0.01) and risk uncontrollability subscale of Metacognition beliefs predicted about 8% of the variance of alexithymia (β=-0.028, P<0.01).

**Conclusions:**

The findings demonstrated that metacognition beliefs and general health had important role in predicting of alexithymia in students.

## 1. Introduction

Emotional development can affect educational achievement. According to one study by (Cherniss & Adler, 2000), general success and welfare in adulthood depends on a) learning how to use social and emotional skills through changes and, b) effective tackling of many life challenges to reduce the risk of mental disorders. In order to facilitate learning, the emotional development of learner must be considered (Greenhalgh, 2002). On the other hand, the context of home with its particular rules and principles; school with the teachers, educational atmosphere and peers, and prevailing culture of relationships will affect emotional development.

Thus, it can be said that one of the significant factors that students struggle with that neglected in educational circles, is inability to express anxieties and emotions (Alexithymia). Alexithymia is a multi-facet construct consisting of difficulty in recognition, description of emotions and distinguishing between emotions and bodily tensions related to emotional excitement and difficulty in expressing feeling for others (Porcelli, Tulipani, Maiello, Cilenti, & Todarello, 2007). Although, individuals with this emotional problem are aware of their emotion experiences, but they can not distinguish the type of emotion. In general population, the prevalence rate of alexithymia reported about 10% that is more prevalent in populations with psychological disorders such as depression, anxiety, pain disorders, sexual disorders, procrastination, substance abuse and educational problems (S. Berthoz, Consoli, Perez-Diaz, & Jouvent, 1999; Sylvie Berthoz, Lalanne, Crane, & Hill, 2013; Dubey, Pandey, & Mishra, 2010a; Hintikka, Honkalampi, Lehtonen, & Viinamäki, 2001; Honkalampi et al., 2010; Thorberg, Young, Sullivan, & Lyvers, 2009).

One of the most important variables related to emotional problems in individuals is metacognition beliefs, beliefs which the person has about his/her thoughts, emotions, memories, feelings and other perceptual forces (Pennebaker, 1997; Saed, Purehsan, Aslani, & Zargar, 2011; Van Dillen & Koole, 2007). These beliefs can influence on person´s response, his/her thoughts, behaviors, emotions and also on self-regulation (Alden, Wiggins, & Pincus, 1990). Metacognitive beliefs are important factors in development and maintenance of psychological disorders. Sometimes, metacognitive beliefs are disrupted and form a set of uncongenial beliefs about internal feelings and thoughts that lead to disturbed regulation (Alden et al., 1990). While knowledge of cognitive and metacognitive strategies lead to improvements in learning and educational performance (Qhavechi & Mohammadkhani Sharam, 2012; Mahasneh, 2014). Also, high levels of impaired metacognitive knowledge have significantly correlated with alexithymia. Furthermore, participants with higher metacognitive knowledge have better educational performance (Sifneos, 1973).

In addition to metacognition, general health status (a state of complete physical, mental and social well-being and not merely the absence of disease or infirmity) (Lorensen, 1978) is factor related to alexithymia. It is assumed that alexithymia can be associated with health. In line with this approach, the researchers found that alexithymia is associated with health problems such as cystitis, essential hypertension, heart disease, diabetes types, depression, anxiety and aggression (Porcelli et al., 2007). According to these findings and important role of metacognitive beliefs and general health on predicting students’ alexithymia, it can be said that inability to express emotions among students demands the attention of authorities, and decision makers in educational systems for reducing emotional impairments in students. Thus, this study attempts to determine the role of metacognitive strategies and general health in predicting alexithymia in high school students.

## 2. Methods

The study was approved by Training and Education Organization of Daregaz and Sari Cities, Iran: (issues 8296 and 35985). In this research after agreement of school administrators, we described the aim of study for students verbally so that they could decide for completing questionnaire themselves. Also they were informed that their all information will remain confidential.

### 2.1 Participants

The study population was all students attending high schools in Daregaz and Sari Cities. Multistage cluster random sampling method was used to 250 participants in the study. Two hundred fifty questionnaire were distributed between participants. Fifty of the questionnaires were not fully completed that they were removed from the research process. Accordingly, at first all high schools in study area were listed. Secondly, two schools from Sari city and two schools from Dargaz city were randomly selected. Finally, two classes were randomly selected from each school. The instruments were used for gathering the data:

### 2.2 Measures

#### 2.2.1 Metacognitive Strategies Questionnaire (MCQ-30)

Metacognitive strategy questionnaire by (A. Wells & Cartwright-Hatton, 2004), consist of 30 items to which each participant can present one of the four options of disagree, somewhat agree, agree, and completely agree. The metacognition is made up of the following sub-scales: a) positive anxiety beliefs, b) beliefs regarding uncontrollability and risk, c) beliefs about cognitive qualifications, d) negative anxiety beliefs and f) Cognitive Self-awareness. These sub-scales are of acceptable reliability and validity. Cronbach’s alpha coefficient of this questionnaire is 0.72 to 0.89 and its test-retest reliability coefficient ranges from 0.76 to 0.80 with the correlation coefficient of the metacognitive questionnaire using anxiety scale of Spiel-Berger between 0.26 and 0.73. (Shirinzadeh Dastgiri, Goudarzi, Rahimi, & Nazari, 2009) has measured Cronbach’s alpha coefficient of this questionnaire for an Iranian sample at 0.91 for the total scale and 0.71 to 0.87 for sub-scales. Furthermore, after four weeks, their test-retest reliability coefficients for total scale were reported 0.73 and for the subscales were 0.58 to 0.87.

#### 2.2.2 Alexithymia Toronto Scale (TAS-20)

(TAS-20), by (Bagby, Parker, & Taylor, 1994) is a self-assessment questionnaire with 20 items used to assess alexithymia. This questionnaire has three dimensions: a) difficulty in diagnosis and detection of emotions (7items); b) difficulty in expressing emotions (5 items) and c) focusing on outside experiences (8 items). The questions are based on the 5-point Likert scale ranging from completely agree =1 to completely disagree =5 (Besharat, 2007). The Cronbach’s alpha coefficient for Alexithymia in Farsi Version was 0.85 and it stood at 0.82, 0.75 and 0.72 for the three sub-scales of difficulty in identifying emotions, difficulty in expressing feelings and extrinsic thinking, respectively, all of which are good internal consistencies (Besharat, 2007). Ghorbani has calculated Cronbach’s alpha coefficient for an Iranian sample population at 0.74 for difficulty in identifying emotions, at 0.61 for difficulty in expression feelings and at 0.50 for extrinsic thinking (Ghorbani, 2002). (Shahgholian, Moradi, & Kafee, 2007) has measured the reliability of the total scale for an Iranian sample using split-half and test-retest at 0.74 and 0.72 respectively, and the validity of the scale at 0.85. The reliability of this questionnaire using Cronbach’s alpha coefficient for the total scale was 0.79.

#### 2.2.3 General Health Questionnaire (GHQ)

The GHQ-28, (Goldberg & Hillier, 1979) is one of the most significant devices for screening mental disorders available as 12, 28, 30 and 60 item lengths. In this study, the 28- item questionnaire was used with its questions containing 4 sub-scales each of 7 items. Questions 1 to 7 are related to the scale of physical symptoms and general health. Questions 8 to 14 are related to the anxiety factor while items 15 to 21 deal with the scale of impaired social performance and 22 to 27 are to do with the depression scale. The present questionnaire is standardized and has been normalized in different populations in Iran and different countries. This questionnaire was simultaneously used with a parallel MHQ test with a correlation coefficient of 55 for the two and correlation coefficients of 0.72 and 0.87 for their subscales that are indicative of high reliability (Taghavi, 2001). This study was done on a sample population of university students and was of high consistency with a Cronbach’s alpha of 0.90 for all items (Taghavi, 2001).

## 3. Results

According to the results shown in table 1, the mean of cognitive confidence (11.92) was higher than other metacognitive strategies. Among the general health variables, the highest mean is for social performance (7.24) and the lowest for physical performance (6.17).

**Table 1:** Mean, standard deviation of study measures ^a^

**Variable**	**Values**
**Alexithymia**	55.28 ± 10.21
**General Health**	
Physical performance	6.17 ± 4.03
Anxiety	7.01 ± 5.34
Social performance	7.24 ± 3.18
Depression	6.35 ± 4.31
**Metacognitive Strategies**	
Positive beliefs about worry	10.29 ± 4.62
Risk Uncontrollability	9.12 ± 4.48
Cognitive Confidence	11.92 ± 4.68
Need to control thoughts	7.56 ± 3.84
Cognitive awareness	6.00 ± 3.54

a Values are presented as mean ± standard deviation.

In order to investigate the relationship between the metacognitive strategies and general health with alexithymia, Pearson’s correlation coefficient was used. As the correlation matrix shows (Table 2), alexithymia is correlated with general health. Physical performance (r=0.287), Anxiety (r=0.360), Social performance (r=0.194) and Depression (r=0.279) had significant positive correlation with alexithymia (P<0.01), and the highest significant correlation was between alexithymia and anxiety subscale (r=0.36, P<0.01). This means that the factors related to health (anxiety level, depression, and physical & social performance) create further tendency to alexithymia. Also, there were negative significant correlations between alexithymia and some metacognitive strategies (risk uncontrollability, cognitive confidence and need to control thoughts) (P<0.05, P<0.01). The highest significant negative correlation was seen between alexithymia and the sub-scale of risk uncontrollability (r=-0.359, *P*<0.01,). There was no significant correlation reported for two sub-scales of positive beliefs about worry (r=-0.085) and cognitive self-awareness (r=-0.035). In order to determine common and specific effects variables (metacognitive strategies and general health) on predicting alexithymia variance, multiple regression was used.

**Table 2:** The results of Pearson’s correlation coefficients between alexithymia, general health and metacognitive strategies subscales ^a^

Variable	1	2	3	4	5	6	7	8	9	10
**Alexithymia**	-									
**General Health**										
Physical performance	0.287^**^	-								
Anxiety	0.360^**^	0.669^**^	-							
Social performance	0.194^**^	0.306^**^	0.321^**^	-						
Depression	0.279^**^	0.506^**^	0.573^**^	0.404^**^	-					
**Metacognitive Strategies**										
Positive beliefs about worry	-0.085	-0.115	-0.053	0.87	-0.090					
Risk uncontrollability	-0.359^**^	-0.370^**^	-0.303^**^	-0.186^**^	-0.360^**^	0.212^**^	-			
Cognitive Confidence	-0.231^**^	-0.213^**^	-0.170^*^	-0.125	-0.286^**^	0.237^**^	0.467^**^	-		
Need to control thoughts	-0.141^*^	-0.125	-0.034	-0.044	0.034	0.158^*^	0.484^**^	0.232^**^	-	
Cognitive awareness	-0.035	-0.173^*^	0.023	0.080	-0.050	0.268^**^	0.341^**^	0.174^*^	0.424^**^	-

a n = 200,

* P < 0.05,

** P < 0.01.

Multiple regression analysis was used to test if metacognitive and general health significantly predicts participants’ alexithymia. Table 3, shows the results of the multistage regression analysis. According to the results of the regression indicate the three predictors explain (21% of the variance (R^2^=0. 21, F=7.238, P < 0.01). Also anxiety subscale of General Health significantly predicts 13% of the variance of alexithymia (β = 0.36, P < 0.01) and risk uncontrollability subscale of Metacognition beliefs predicts about 8% of the variance of alexithymia (β=-0.028, P < 0.01).

**Table 3:** The result of multiple regressions for prediction alexithymia according to metacognitive beliefs and general health subscales

Variables	*β*	*t*	*P*	*F*	*R*	*R^2^*	*Adjusted R^2^*
**Model 1**							
Anxiety	0.360	5.431	0.000	29.493	0.360	0.130	0.125
**Model 2**							
Anxiety	0.304	3.405	0.000	15.181	0.365	0.134	0.125
Physical Performance	0.084	0.941	0.001				
**Model 3**							
Anxiety	0.262	2.730	0.007	10.549	0.374	0.140	0.126
Physical Performance	0.064	0.696	0.487				
Depression	0.097	1.167	0.245				
**Model 4**							
Anxiety	0.261	2.710	0.007	8.036	0.377	0.142	0.124
Physical Performance	0.060	0.652	0.515				
Depression	0.072	0.804	0.423				
Social Performance	0.057	0.753	0.453				
**Model 5**							
Anxiety	0.259	2.791	0.006	9.997	0.454	0.206	0.185
Physical Performance	-0.010	-0.116	0.907				
Depression	0.007	0.085	0.932				
Social Performance	0.059	0.798	0.426				
Risk Uncontrollability	-0.278	-3.931	0.000				
**Model 6**							
Anxiety	0.263	2.834	0.005	7.238	0.458	0.210	0.181
Physical Performance	-0.012	-0.129	0.897				
Depression	-0.005	-0.054	0.957				
Social Performance	0.060	-0.820	0.413				
Risk Uncontrollability	-0.247	-3.190	0.002				
Cognitive Confidence	-0.072	-0.977	0.330				
**Model 7**							
Anxiety	0.263	2.825	0.005	0.263	0.458	0.210	0.181
Physical Performance	0.012	-0.128	0.899				
Depression	-0.005	-0.057	0.955				
Social Performance	-0.060	0.817	0.415				
Risk Uncontrollability	-0.248	-2.821	0.005				
Cognitive Confidence	-0.072	-0.975	0.331				
Need to control thoughts	0.002	0.021	0.983				

## 4. Discussion

The results of regression analysis showed that anxiety and uncontrollability of risk are the most important variables in predicting alexithymia. These findings are consistent with research results reported by some researchers: (Bucci, 1997; Dubey, Pandey, & Mishra, 2010b; Garnefski & Kraaij, 2006; Mikhaeili, Karimnejad, Irani, & Pirnabikhah, 2012; Porcelli et al., 2007; Qhavechi & Mohammadkhani Sharam, 2012; Z. Rostam Oghali, Mosazadeh, Rezazade, & S. Rostam Oghali, 2013).

It seems that in accounting for the findings of this study the interaction between metacognitive strategies, general health and alexithymia must be considered. The alexithymia refers to failure of individual to identify and express the feelings and distinguishing them from bodily sensations, paucity of fantasies, and an externally oriented cognitive style (Taylor & Bagby, 2000; Taylor, Bagby, & Parker, 1999). This inability can’t disrupts the individual’s emotional and cognitive organization and increases the probability of utilizing inefficient styles. Deficiency in emotional regulations will lead to negative experiences. As a result, when people understand that their control over the circumstances have diminished, they interpret the situation as stressful and negative condition; which in turn would lead to negative emotions and interpersonal issues like cold relationships (Bailey & Henry, 2007). In addition, it can be noted that inability to regulate emotions can be related to low levels of physical and mental health. Health is a multi-dimensional concept. Attending this issue and attempting to recognize their consistent impacts on one another are essential (Sharepour, 2002). According to Shahgholian, Moradi and Kafee (2007), emotionally potent people are better adapted with the environment and others in comparison with those who fail to grasp their emotional excitements. Thus, according to the previous studies it can be said that those who are more potent in experiencing and expressing emotions are of healthier than others. Also findings of other studies (Groh, Jason, & Keys, 2008; Hendriks, 1990; Maremmani, Pani, Pacini, & Perugi, 2007; Shahgholian M. et al., 2007) approve this conclusion that alexithymia is due to problems in identifying and adjusting emotions.

The current study showed that there is a positive significant correlation between metacognition and alexithymia and the findings of this study are consistent with earlier reports (Babaei, Gharechahi, Hatami, & Ranjbar Varandi, 2015; Hoffman, Bobby, Spatariu, & Alexandru, 2008; Molavi, Rostami, Fadaee Naeini, Mohamadnia, & Rasolzadeh, 2007; Rostam Oghali et al., 2013; Zhu & Leung, 2011). In agreement with these findings it can be said that the metacognition is an important variable that affects person’s response to negative thoughts, signs and emotions (Adrian Wells, 2002, 2011). Since metacognitive knowledge encompasses a person’s emotions and experiences and regulates the mind and method of studying; thus it enables the person to guide his/her thought in learning and problem solving situations using mastery of learning and studying principles leading to better memory performance (Larkin, 2009). Hence, it can be concluded that knowledge of and exercising metacognition can significantly influence different aspects of a student’s life. Thus by knowing this a person can use it while exhibiting a more comprehensive control, selecting the most efficient strategy (Artino, 2008). Finally, similar to other studies current research has some inherent limitations, and it is suggested that these limitations must be considered while generalizing its results. The sample population was limited to students and in generalization of results to other populations must be considered. Also it is suggested that alexithymia be investigated in other mental disorders. The results of the present study provided the effects of metacognitive strategies and general health on alexithymia; therefore it is essential that experts consider investigating this field and extend it to other areas.

## 5. Conclusion

These findings demonstrated that metacognition beliefs and general health had important role in predicting of alexithymia in students

## Authors’ Contribution

Samaneh Babaei has written whole of the manuscript and Shahryar Ranjbar Varandi; Zohreh Hatami and, Maryam Gharehchahi have collected data.
